# Validation of age, PaO_2_/FlO_2_ and plateau pressure score in Korean patients with acute respiratory distress syndrome: a retrospective cohort study

**DOI:** 10.1186/s12931-020-01357-5

**Published:** 2020-04-22

**Authors:** Hyeontaek Hwang, Sun Mi Choi, Jinwoo Lee, Young Sik Park, Chang-Hoon Lee, Chul-Gyu Yoo, Young Whan Kim, Sung Koo Han, Sang-Min Lee

**Affiliations:** Division of Pulmonary and Critical Care Medicine, Department of Internal Medicine, Seoul National University Hospital, College of Medicine, Daehak-ro 101, Jongro-gu, Seoul, 03080 Republic of Korea

**Keywords:** Respiratory distress syndrome, Adult, Prognosis, Hospital mortality, ROC curve, Calibration

## Abstract

**Background:**

A predictive scoring system for acute respiratory distress syndrome (ARDS) patients, which incorporates age, PaO_2_/FlO_2_, and plateau pressure, APPS, was developed recently. It was validated externally in a Caucasian population but has not been studied in Asian populations. The aim of this study was to validate APPS in Korean ARDS patients.

**Methods:**

We retrospectively reviewed the medical records of patients who were diagnosed with ARDS using the Berlin criteria and admitted to the medical ICU at Seoul National University Hospital from January 2015 to December 2016. The validation of the APPS was performed by evaluating its calibration and predictive accuracy. Its calibration was plotted and quantified using the Hosmer–Lemeshow test. Its predictive accuracy was assessed by calculating the area under the receiver operating characteristics (AUC–ROC) curve.

**Results:**

A total of 116 patients were analyzed, 32 of whom survived. Of the 116 patients, 11 (9.5%) were classified as APPS grade 1 (score 3–4), 88 (75.9%) as grade 2 (score 5–7) and 17 (14.6%) as grade 3 (score 8–9). In-hospital mortality was 27.3% for grade 1, 73.9% for grade 2 and 94.1% for grade 3 (*P* for trend < 0.001). The APPS was well calibrated (Hosmer–Lemeshow test, *P* = 0.578) and its predictive accuracy was acceptable (AUC–ROC 0.704, 95% confidence interval 0.599–0.809).

**Conclusions:**

The APPS predicted in-hospital mortality in Korean patients with ARDS with similar power to its application in a Western population and with acceptable predictive accuracy.

**Trial registration:**

Retrospectively registered.

## Background

Since the initial description of acute respiratory distress syndrome (ARDS) in 1967 [1], its definition has been clarified by expert consensus [2, 3], and epidemiological studies have demonstrated its clinical significance in intensive care units (ICUs) [4–6]. A recent multicenter observational cohort study, in which ARDS was defined according to the Berlin criteria, reported that the prevalence of ARDS was 10.4% at ICU admission and that its overall hospital mortality was 40.0% [4]. Because ARDS has a high prevalence and mortality in ICU, it is important to determine a precise prognosis that allows clinicians to predict the clinical course of ARDS and decide on a treatment plan. To do this consistently and reasonably, a scoring system is required.

There has been no reliable prognostic scoring system for ARDS, despite the existence of prognostic indices such as the Acute Physiology and Chronic Health Evaluation (APACHE II), Simplified Acute Physiology Score (SAPS II), and Sequential Organ Failure Assessment (SOFA) [7–9]. Although these have been used to predict general mortality in ICU patients, they are not specific for ARDS. In addition, they involve complex calculations that require extensive clinical information about the patients.

Recently, a new, simpler prognostic scoring system was developed that is specific for ARDS: the age, PaO_2_/FlO_2_, and plateau pressure score (APPS). The APPS is a 9-point score that is calculated by measuring the age, PaO_2_/FIO_2_ ratio, and plateau pressure at 24 h after the patient is diagnosed with moderate to severe ARDS and counting each one to 1–3. Since many variables are not needed for calculations, clinicians can easily predict the in-hospital mortality of mechanically ventilated patients with moderate to severe ARDS patients at bedside [10]. However, one of the limitations of APPS was that it was derived and validated in cohorts from a number of Spanish hospitals and one American hospital. It has not been validated in an Asian population. Few studies have examined the geographic variation in the incidence rate of ARDS as defined by the Berlin criteria, and it remains unclear whether the features of ARDS in Asia differ from those in Western countries [4, 11]. In this context, we aimed to validate APPS externally for mechanically ventilated ARDS patients in Korea and assess the differences between Asian and Western populations.

## Methods

We retrospectively reviewed patients admitted to the medical ICU at Seoul National University Hospital (SNUH) from January 1, 2015 to December 31, 2016. The study included patients who were mechanically ventilated in the medical ICU for at least 24 h and were confirmed to have moderate or severe ARDS according to the Berlin criteria [3]. Patients in a ward or ICU had respiratory failure within a week and started receiving mechanical ventilation after intubation in the ICU. They had bilateral opacity in chest imaging, proved not to be due to cardiac failure or fluid overload, which was evaluated by transthoracic echocardiography if necessary. In the ventilator setting, the peak end-expiratory pressure (PEEP) was 5 cmH_2_O or above. The PaO_2_/FIO_2_ ratio of the patients was 200 mmHg or below. Although patients were not treated according to strict protocols, it was recommended that patients received lung-protective mechanical ventilation, i.e., were ventilated with a low tidal volume of 4–8 mL/kg predicted body weight. The study excluded patients who were not intubated within 24 h after ARDS diagnosis because the calculation of APPS requires measurement of the plateau pressure. It also excluded patients who died or were discharged less than 24 h after diagnosis.

We collected information on the baseline characteristics of the patients, including demographic characteristics, underlying comorbidities, and the cause of ARDS. SAPS II, APACHE II, and SOFA scores were used to assess the severity of the patients’ general condition. We also collected arterial blood gas analysis data and ventilator-related indices for patients, and assessed whether they underwent adjuvant therapies such as the use of nitric oxide gas, prone positioning, or extracorporeal membrane oxygenation (ECMO). The primary clinical outcome was in-hospital mortality. The assessment of in-ICU mortality and the length of stay in ICU were used as secondary clinical outcomes.

The APPS was calculated and divided into three grades as defined in the original report [10]. Since the APPS was calculated at 24 h after ARDS diagnosis, we read age, PaO_2_/FIO_2_ ratios and maximal airway pressure at 24 h after ARDS diagnosis. When several measurements were available, the measurements taken at the closest time 24 h after diagnosis were selected. Grade 1 was defined as a score of 3–4 points, grade 2 as 5–7 points and grade 3 as 8–9 points. However, one difference was that this study used the maximal airway pressure rather than the plateau pressure because almost all of the patients in SNUH ICU received pressure-controlled mechanical ventilation. Under most conditions, maximal airway pressure during pressure-controlled ventilation is roughly equivalent to plateau pressure during volume-controlled ventilation.

Descriptive data were expressed as mean with standard deviation, median with interquartile range, or number with percentage, depending on the variable. Student’s *t* test, one-way analysis of variance, Mann–Whitney *U* test, or the Kruskal–Wallis test were used to compare continuous variables, and Pearson’s chi-squared test, Fisher’s exact test, or linear-by-linear association were used to compare categorical variables. Pearson and Spearman correlation analysis were used to determine the correlation between the two variables. To validate the predictive performance of APPS, we evaluated its quantitative calibration and discriminatory ability [12]. We compared the predicted and observed in-hospital mortality using a calibration plot and the Hosmer–Lemeshow goodness-of-fit test. The discrimination was measured by calculating the C-statistics from the receiver operating characteristics (ROC) curve. The probability of survival of all patients was analyzed using the Kaplan–Meier method with the log-rank test. The data for patients who survived and were discharged were censored at the last hospital follow-up date. A sensitive analysis was performed on patients who received mechanical ventilation with the standardized ventilatory setting (FIO_2_ ≥ 0.5; PEEP ≥10cmH_2_O) in the derivation study of APPS [10].

## Results

**Fig. 1:**
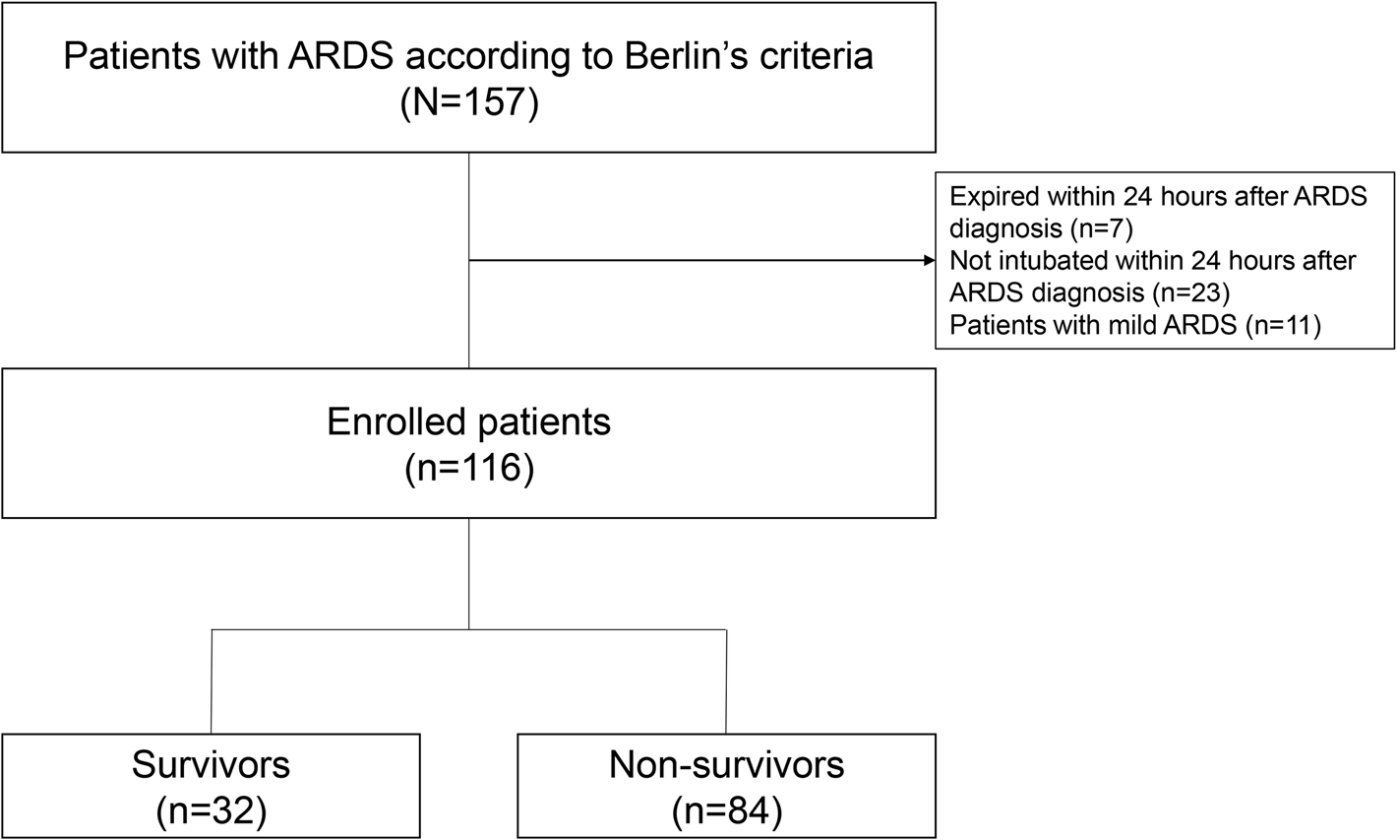
Flow chart

Of the 157 patients diagnosed with ARDS according to the Berlin criteria, 116 were analyzed, excluding patients with mild ARDS and those who died or were not intubated within 24 h after diagnosis of ARDS (Fig. 1). Of these 116 patients, 32 survived, and we compared the baseline characteristics of the survivors and nonsurvivors (Table 1). There was no significant difference between survivors and nonsurvivors in sex, age, underlying disease, cause of ARDS, physiologic parameters, or ventilatory parameters. The median APPS of survivors and nonsurvivors were 5.0 (interquartile range 4.3–6.0) and 6.0 (interquartile range 5.0–7.0), respectively; the APPS of nonsurvivors was significantly higher than that of survivors (*P* < 0.001). In addition, the mean APACHE II scores of survivors and nonsurvivors were 31.8 ± 8.5 and 35.2 ± 8.1, respectively, with that of nonsurvivors also significantly higher than that of survivors (*P* = 0.047).

**Table 1:** Baseline characteristics of the survivors and non-survivors with acute respiratory distress syndrome^a^

	Survivor(***n*** = 32)	Non-survivor(***n*** = 84)	***p***-value
Male, N(%)	24 (75.0)	57 (67.9)	0.454
Age, mean ± SD	64.5 ± 17.1	66.7 ± 13.2	0.459
**Underlying disease, N(%)**			
Diabetes	7 (21.9)	24 (28.6)	0.466
Hypertension	12 (37.5)	26 (31.0)	0.502
Tuberculosis	1 (3.1)	10 (11.9)	0.285
Chronic liver disease	1 (3.1)	5 (6.0)	1.000
**Cause of ARDS, N(%)**			
Pneumonia	25 (78.1)	63 (75.0)	0.812
Aspiration	3 (9.4)	5 (6.0)	0.683
Sepsis	3 (9.4)	8 (9.5)	1.000
Transfusion	0 (0)	1 (1.2)	1.000
Others	1 (3.1)	7 (8.3)	0.442
**Disease severity, mean ± SD**			
SAPS II	75.0 ± 22.0	79.0 ± 19.4	0.338
APACHE II	31.8 ± 8.5	35.2 ± 8.1	0.047
SOFA	13.7 ± 4.0	13.6 ± 3.9	0.874
APPS, median ± IQR	5.0, 4.3–6.0	6.0, 5.0–7.0	< 0.001
**Physiological parameters, mean ± SD**			
pH, median ± IQR	7.4, 7.3–7.4	7.3, 7.3–7.4	0.066
PaCO_2_	43.5 ± 14.6	45.8 ± 13.6	0.430
PaO_2_/FiO_2_	111.5 ± 40.8	103.4 ± 36.0	0.294
**Ventilator parameters, mean ± SD**			
Tidal volume (mL/kg PBW)	7.1 ± 2.1	7.5 ± 2.6	0.503
Minute ventilation(L/min)	10.4 ± 3.5	10.0 ± 3.5	0.649
FIO_2_	0.76 ± 0.18	0.82 ± 0.19	0.166
PEEP (cmH_2_O)	7.2 ± 2.4	7.3 ± 2.6	0.859
P_max_ (cmH_2_O)	23.7 ± 4.9	24.7 ± 4.8	0.298

*Abbreviation*: *APACHE* acute physiology and chronic health evaluation, *APPS* age, PaO_2_/FlO_2_, and plateau pressure score, *IQR* interquartile range, *SAPS* simplified acute physiology score, *SOFA* sequential organ failure assessment;

^a^ Data are presented as n (%) or mean ± SD or median ± IQR

**Fig. 2:**
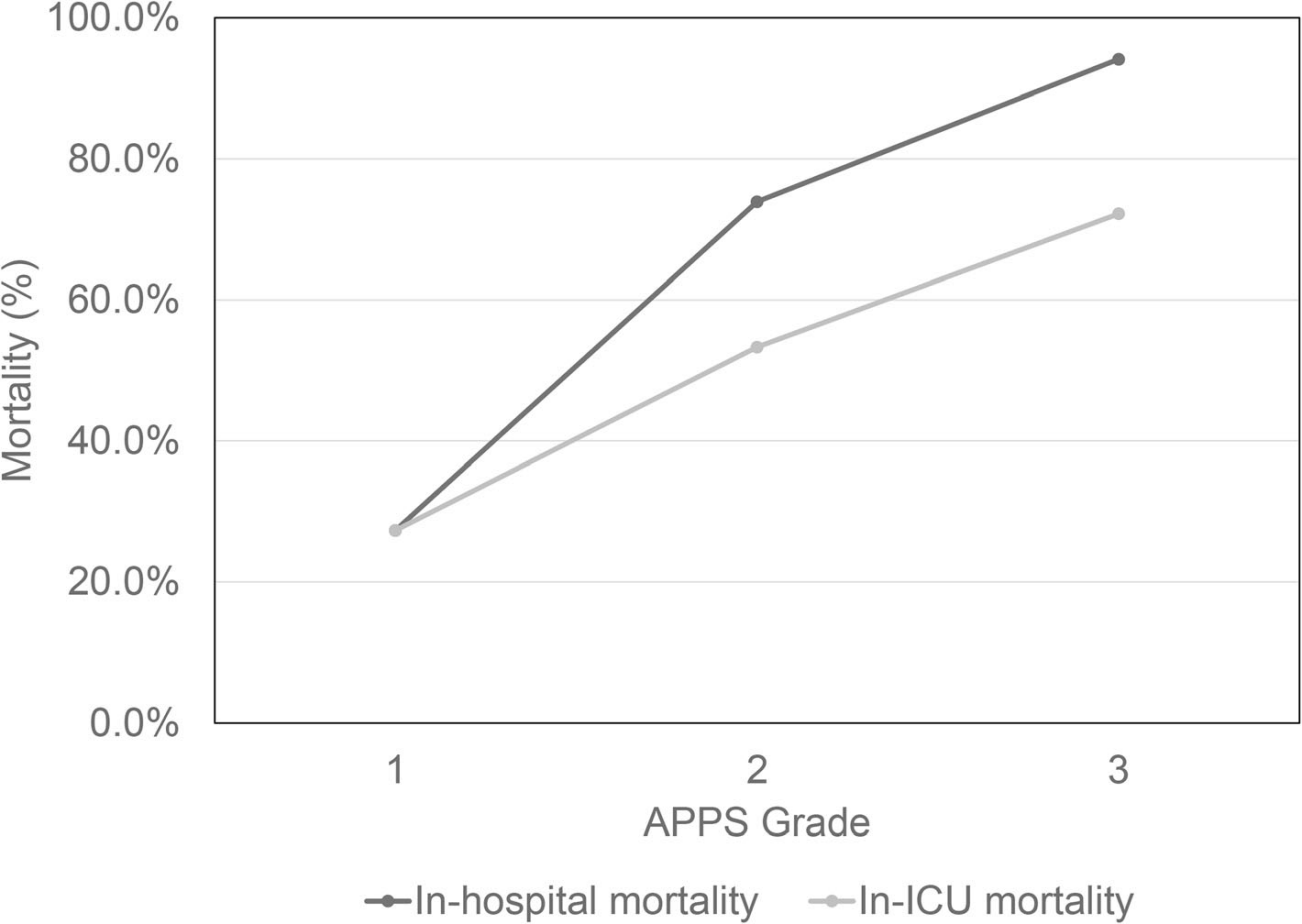
Mortality according to APPS grade. In-hospital mortality, p for trend < 0.001, In-ICU mortality, p for trend = 0.028

As the APPS grade increased, in-hospital mortality and in-ICU mortality increased significantly (Table 2, Fig. 2), the frequency of extubation was significantly reduced, and the use of nitric oxide significantly increased. Other clinical outcomes did not differ significantly according to APPS grade. Of the enrolled patients, 8 (6.9%) patients received ECMO treatment and 18 (15.5%) patients received prone position. In the patients who received ECMO treatment, except for one patient whose APPS was 3, all patients had an APPS score of 5 or higher (APPS Grade ≥ 2). There was a correlation between the APACHEII score and APPS, but it was not statistically significant. (Pearson’s correlation coefficient 0.621, *P* = 0.100) In the patients who received prone position, except for two patients whose APPS was 4, all patients had an APPS score of 5 or higher (APPS Grade ≥ 2). There was a significant correlation between the APACHEII score and APPS. (Spearman’s correlation coefficient 0.502, *P* = 0.034).

**Table 2:** Clinical outcomes according to APPS grade^a^

	APPS Gr 1 (***n*** = 11)	APPS Gr 2 (***n*** = 88)	APPS Gr 3 (***n*** = 17)	***p*** for trend
MV duration, median (IQR), days	10 (3–23)	10 (5–21)	12 (6–21)	0.721
Extubation, N(%)	7 (63.6)	33 (37.5)	3 (17.6)	0.015
Tracheostomy, N(%)	2 (18.2)	21 (23.9)	4 (23.5)	0.787
Hospital length of stay, median (IQR), days	27 (17–73)	27 (15–52)	21 (13–51)	0.694
ICU length of stay, median (IQR), days	11 (3–23)	12 (6–22)	13 (8–21)	0.702
In-hospital mortality, N(%)	3 (27.3)	65 (73.9)	16 (94.1)	< 0.001
In-ICU mortality, N(%)	3 (27.3)	47 (53.4)	12 (70.6)	0.028
Use of adjuvant therapy, N(%)				
Inotropics	9 (81.8)	79 (89.8)	15 (88.2)	0.687
Corticosteroid	10 (90.9)	81 (92.0)	17 (100)	0.291
Nitric oxide	4 (36.4)	42 (47.7)	13 (76.5)	0.024
Prone position	2 (18.2)	15 (17.0)	1 (5.9)	0.313
CRRT	2 (18.2)	32 (36.4)	4 (23.5)	0.989
ECMO	1 (9.1)	6 (6.8)	1 (5.9)	0.757

*Abbreviation*: *APPS* , PaO_2_/FlO_2_, and plateau pressure score, *CRRT* continuous renal replacement therapy, *ECMO* extracorporeal membrane oxygenation, *ICU* intensive care unit, *MV* mechanical ventilation;

^a^ Data are presented as n (%) or mean ± SD or median ± IQR

**Fig. 3:**
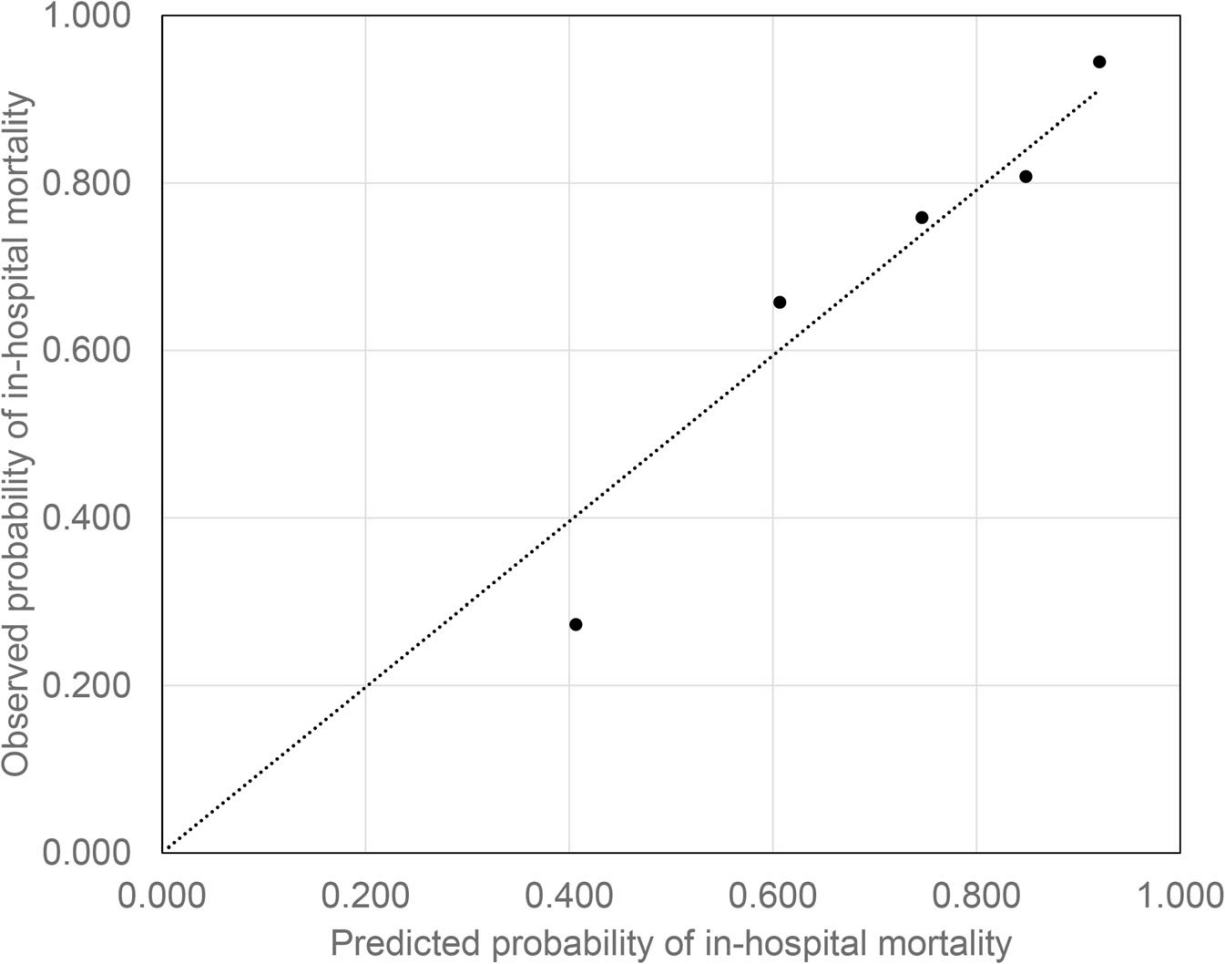
Calibration plot of APPS

**Fig. 4:**
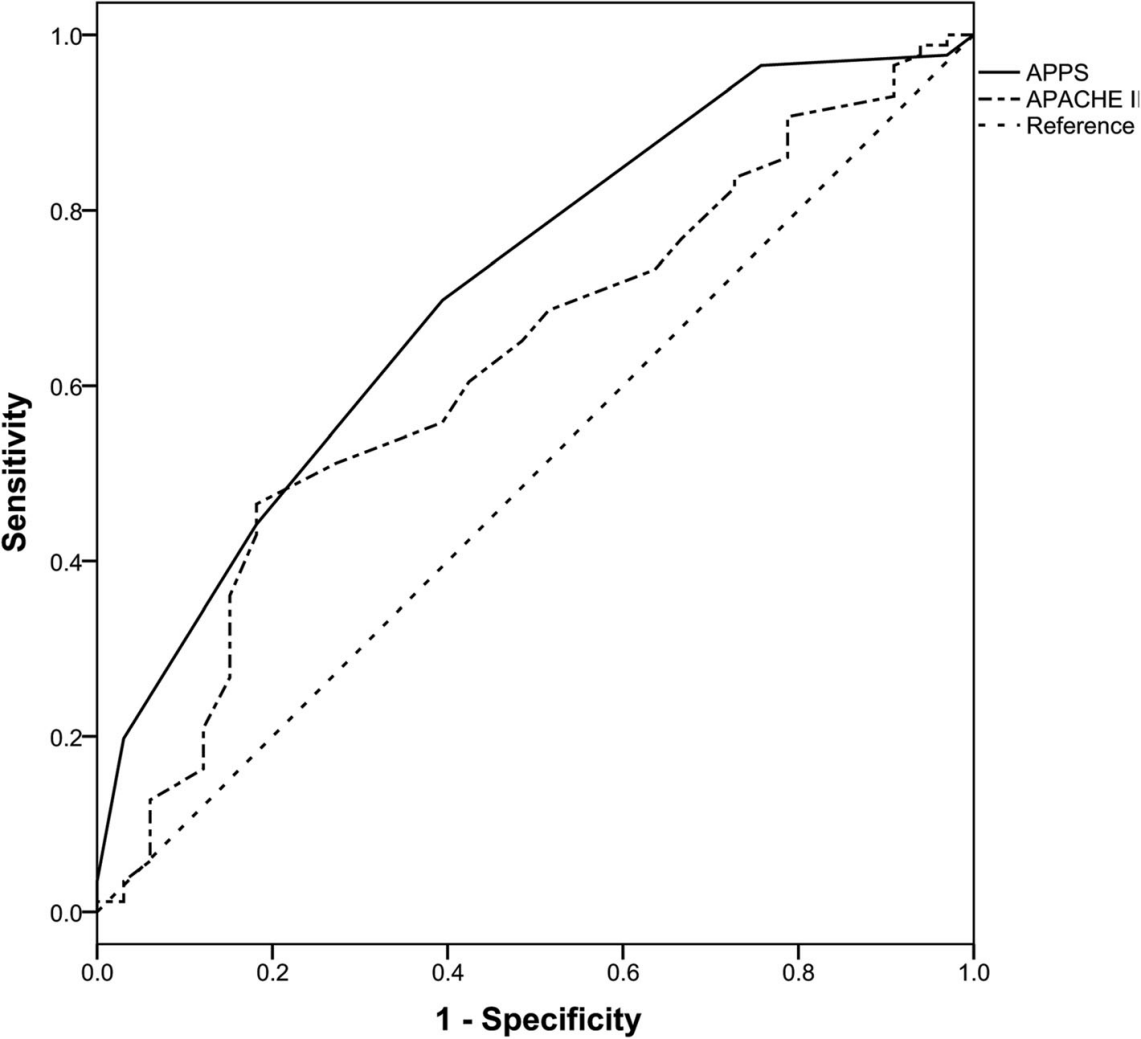
Receiver operating characteristic curve of APPS and APACHE II. APPS (c-statistics 0.704, 95% CI [0.599–0.809]), APACHE II (c-statistics 0.623, 95% CI 0.510–0.736)

**Fig. 5:**
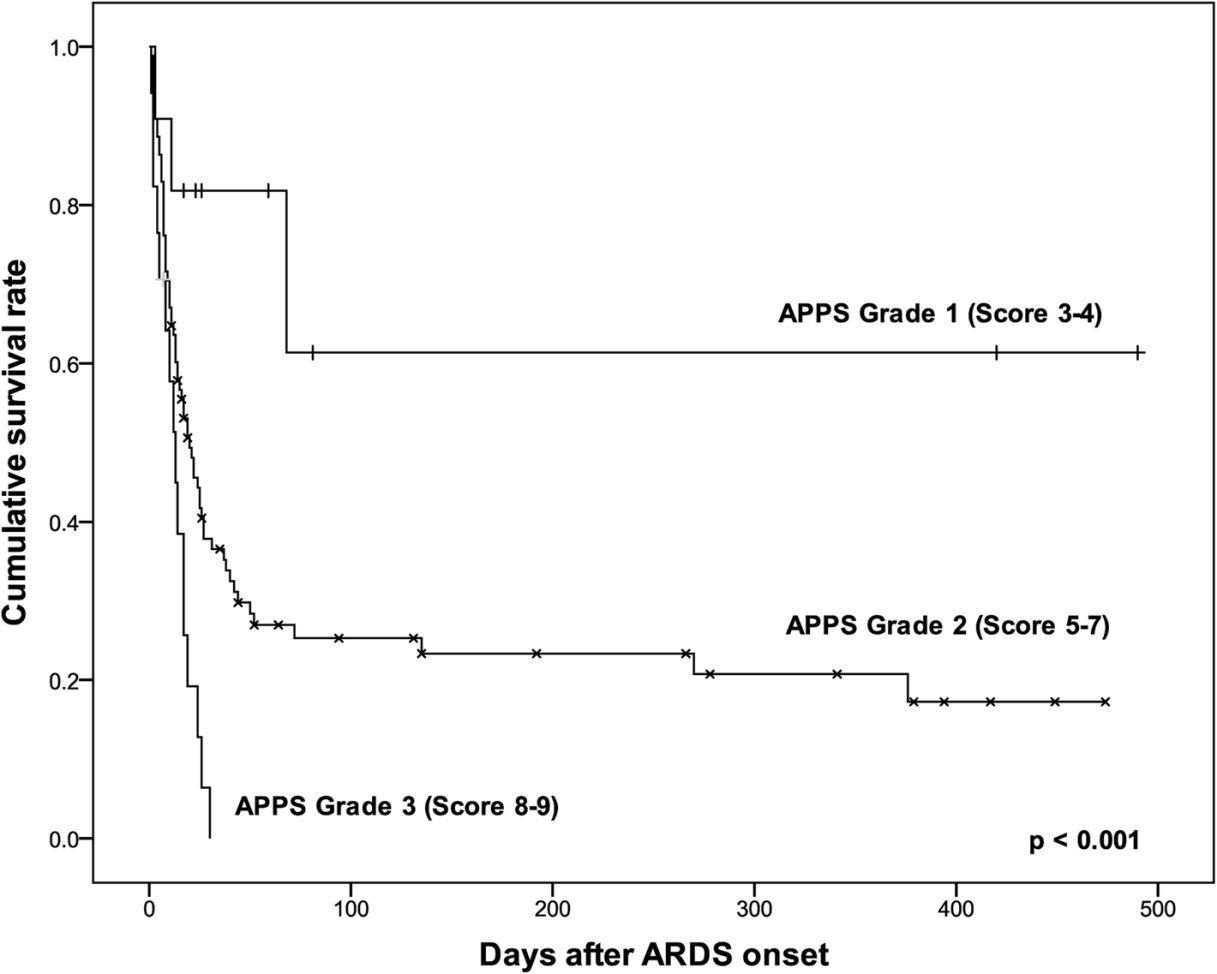
Kaplan-Meier probability of survival curves according to APPS grade

The probability of in-hospital death predicted by the APPS model and the observed rate of in-hospital death were compared using the calibration plot and the Hosmer–Lemeshow goodness-of-fit test (Fig. 3). The APPS model was well calibrated and there was no significant difference between the predicted and observed probability of in-hospital death (*P* = 0.639). To verify the accuracy of the APPS model and to compare it with APACHE II, ROC curves were generated for the two scoring systems (Fig. 4). The APPS model had fair accuracy: the C-statistic for the APPS model obtained from the area under the ROC curve was 0.711 (95% confidence interval [CI] 0.609–0.813), which was higher than that of APACHE II (0.624; 95% CI 0.513–0.736). When the Kaplan–Meier curve of patient survival was drawn, the cumulative survival rate clearly differed according to APPS grade (Fig. 5). The probability of survival decreased as the APPS grade increased (*P* < 0.001).

A sensitive analysis (*n* = 26) was conducted on patients who received mechanical ventilation with the standardized ventilatory setting (FIO_2_ ≥ 0.5; PEEP ≥10cmH_2_O), following the ventilatory protocol in the derivation study of APPS. The APPS model was confirmed to be well calibrated by the Hosmer-Lemeshow goodness-of-fit test (*P* = 0.647). This analysis showed that the predictive accuracy of the APPS was excellent. (AUC–ROC 0.823, 95% CI 0.651–0.996).

## Discussion

This study showed that APPS was well calibrated and had good predictive accuracy for the prognosis of moderate to severe ARDS in Asian populations. The original report that proposed APPS stated that the C-statistic of APPS was 0.755 (95% CI 0.699–0.811) in the derivation cohort, and 0.800 (95% CI, 0.750–0.850) in the validation cohort [10]; the C-statistic of APPS for the present study was similar to that of the original study. In contrast, in a spatial validation study conducted in the Netherlands [13], APPS was not well calibrated, with a relatively low C-statistic value of 0.62 (95% CI 0.56–0.67), indicating a relatively low predictive accuracy. There are several possible reasons for the difference between these two external validation results. First, there was a difference in the severity of the illness in the patients. In the present study, the hospital mortality rate was 72.3% and the mean APACHE II score of all patients was 34.3 ± 8.3, both higher than those in the Dutch study, in which APPS was not effective in predicting mortality in the group with low scores. Similarly, in our study, patients with an APPS score of 3, which is the lowest possible score, had relatively high mortality but decreased APPS predictive accuracy. This suggests that APPS may be more accurately predictive in patients with more severe disease. Second, there may be ethnic differences in the validation of APPS. Although there are no previous studies of the relationship between Asian ethnicity and ARDS mortality, a retrospective study reported that African–American and Hispanic patients with ARDS had a significantly higher risk of death than Caucasian patients [14]. Another study reported that although African–American patients had more severe clinical manifestations of ARDS than Caucasian patients, their incidence of ARDS was lower [15]. ARDS is a complex clinical syndrome involving various pathophysiologic mechanisms, and environmental and genetic factors are implicated in its development and progression [16]. There has been no report identifying specific genetic polymorphisms that affect the prognosis of ARDS in Asians, but some studies have shown that genetic polymorphisms associated with inflammation, innate immunity, epithelial cell function, and angiogenesis are related to the prognosis of ARDS [17–22]. Therefore, the heterogeneity of ethnicity-related factors in ARDS may have affected the predictive accuracy of the APPS.

As reported in the original article describing APPS and the Dutch study discussed above, the present study also found that the ability of APACHE II scores to predict the prognosis of moderate to severe ARDS was inferior to that of APPS. One study reported that the APACHE II score was a predictor of mortality in ARDS, but that it was less relevant than other indicators such as age [23]. The APACHE II score is a general scoring system for ICU patients and may be less predictive when applied to ARDS patients. The Murray lung injury score for assessing the severity of acute lung injury was suggested in 1988 [24], and the CESAR study showed that ECMO treatment improved survival in ARDS patients with severe respiratory failure (Murray score > 3 or pH < 7.20) [25]. However, the Murray score has been criticized for its lack of specificity and validation in ARDS patients [26]. In contrast, APPS is specific for predicting the prognosis of ARDS and can be calculated easily, and thus can be used in clinical practice.

The sensitive analysis was performed on patients who received mechanical ventilation with the standardized ventilatory setting used in the derivation study of APPS because the PaO_2_/FIO_2_ ratio may be affected by the ventilator setting [27]. In the analysis, the APPS also was well calibrated and showed good predictive accuracy.

This study has several limitations. First, there may be a selection bias because it was a single-center retrospective study. Many of the patients had various underlying diseases, including malignancy, because of the characteristics of the tertiary hospital in Korea. Only a small number of patients had a low APPS because the general condition of most of the patients was very severe. Nevertheless, this study confirmed that APPS was as valid in Asian patients as it was in the original study in Western countries, and because of its specificity and simplicity, could be used in the treatment and research of ARDS patients in Asian countries. However, a multicenter prospective cohort study in Asia is needed to produce more reliable results. Second, the present study validated APPS differently from the original study by using maximal airway pressure instead of plateau pressure. We had difficulty monitoring plateau pressure in our patients because they all received pressure-controlled mechanical ventilation. However, despite these differences in validation procedure, this study showed that APPS could be appropriately applied using maximal airway pressure instead of plateau pressure. In the external validation study conducted in the Netherlands, it was also performed using maximal airway pressure instead of plateau pressure [13]. This suggested that APPS is more clinically useful than other scoring systems for predicting the prognosis of ARDS because, in clinical practice, the protocol for mechanical ventilation therapy may be different for each ICU.

## Conclusions

The APPS predicted in-hospital mortality with acceptable predictive accuracy in Korean patients with ARDS similarly to Western populations.

## Data Availability

The datasets used and/or analysed during the current study are available from the corresponding author on reasonable request.

## Abbreviations

APACHE: Acute physiology and chronic health evaluation
APPS: Age, PaO_2_/FlO_2_, and plateau pressure score
ARDS: Acute respiratory distress syndrome
AUC–ROC: The area under the receiver operating characteristics curve
ECMO: Extracorporeal membrane oxygenation
ICU: Intensive care unit
PEEP: Peak end-expiratory pressure
SAPS: Simplified acute physiology score
SNUH: Seoul National University Hospital
SOFA: Sequential organ failure assessment
ROC: Receiver operating characteristics
